# Menstruation practice among school and out-of-school adolescent girls, Lao PDR

**DOI:** 10.1080/16549716.2020.1785170

**Published:** 2020-08-03

**Authors:** Vanphanom Sychareun, Kongmany Chaleunvong, Dirk R. Essink, Phouthong Phommavongsa, Joanne Durham

**Affiliations:** a Faculty of Public Health, University of Health Sciences, Vientiane, Lao PDR; b Institute of Research and Education Development, University of Health Sciences, Vientiane, Lao PDR; c Athena Institute for Research on Innovation and Communication in Health and Life Sciences, Faculty of Earth and Life Sciences, Vrije Universiteit, Amsterdam, Netherlands; d School of Public Health and Social Work, Queensland University of Technology, Brisbane, Australia

**Keywords:** LEARN: Sexual Reproductive Health, ANC and Nutrition, Menstruation, menstrual hygiene management, adolescent girls, menarche, attitudes

## Abstract

**Background:**

The transition from childhood to adolescence is a critical transitional period for girls, and as they experience these changes, having knowledge of, and being able to practice, good menstruation hygiene management is crucial. The objective of this study was to understand in and out of school adolescents’ menstrual hygiene management practice, sources of information and attitudes towards menstruation.

**Methods:**

A cross sectional, descriptive study was conducted between November 2018 and May 2019. The sampling included adolescent girls aged 11–19 years in higher secondary schools in Khammouane and Champassak provinces and out-of-school adolescent girls. Out of the total number of 433 participants enrolled in the study, only 343 girls had reached menarche and were included in the study. Factors associated with menstruation hygiene management practice were examined with bivariate and multivariate analyses.

**Results:**

Out of 343 subjects who had reached menarche, 44% reported good menstrual hygiene management practice. Over half of the participants, however, were unable to access the tools of good menstruation hygiene management practice, including having somewhere to dispose of used sanitary pads in private. Being older (16–19 years) (AOR:2.4; 95%CI 1.4 to 3.9), and having primary education (AOR 0.4; 95%CI 0.2 to 0.9) were associated with good practice as was fathers’ level of education (AOR 2.1; 95%CI 1.0 to 4.1) and mother-girl discussion about menstruation (AOR 2.2; 95%CI 1.0 to 5.0). No significant differences were found between in- and out-of-school adolescents.

**Conclusions:**

In this study, not all participants were able to practice optimal menstruation hygiene management. This is important as good menstrual hygiene management is associated with better health and being able to participate fully in education and work. Further education is needed, but it is also important to ensure that all adolescent girls have access to the necessary tools for effective menstruation hygiene management.

## Background

The transition from childhood to early adolescence is a critical period characterised by the onset of puberty and rapid physical and psychosocial changes [1]. For girls, puberty begins with thelarche, accelerated growth and the onset of menarche, which marks the beginning of the reproductive phase of a woman’s life [2]. Menarche is a natural physiological process and a sign of good physical health, yet despite these many girls, especially in lower- and middle-income countries (LMICs), start their menstruation unprepared for managing their periods and unsure of when and where to seek help [3-7]. Most adolescent girls turn to their mothers or other female confidantes to learn about menstruation, but these female confidantes themselves may be misinformed and uncomfortable in discussing sexuality, reproduction and menstruation [1,6–8]. Addressing knowledge gaps is important as shame, emotional distress and poor menstrual hygiene management (MHM) have been associated with a lack of knowledge [8]. While most adolescent girls turn to their mothers or other female confidantes to learn about menstruation, these female confidantes may themselves be misinformed or uncomfortable in discussing sexuality, reproduction and menstruation [2,9–11]. Knowledge gaps in relation to menarche are important; however, with studies suggesting where girls feel unprepared for menarche, they are more likely to experience distress at menarche compared with those who feel well-prepared and experience more painful menstrual symptoms.

Inadequate knowledge and understanding of menstruation are of concern because these can be harmful to adolescents’ mental and physical health. Where adolescent girls have knowledge gaps related to menarche, and their menstrual cycle, they can experience feelings of shame and distress. The stigmatized nature of menstruation and taboos around discussing sexuality and the need for concealment can also lead to girls internalizing menstrual stigma. Knowledge gaps can prevent effective Menstrual Hygiene Management (MHM), defined as women and adolescent girls using clean menstrual management material to absorb or collect blood, that can be changed in privacy as often as necessary during the menstrual period, using soap and water for washing the body as required, and having access to facilities to dispose of used menstrual management materials. [12] Suboptimal MHM is associated can cause genital discomfort, irritation, rashes, and bruising during menses due to the quality of menstrual materials or not changing menstrual pads frequently enough [10,13–15]. Poor MHM practices are also associated with increased risk of reproductive and urinary tract infections, cervical cancer and adverse pregnancy outcomes [16,17].

Additionally, inadequate MHM practices are linked to reduced academic performance and school attendance, with potential impacts on longer term socio-economic outcomes and overall poor quality of life [2,5,13,18,19]. Even where females have a good understanding of MHM, they may lack access to the facilities and products needed to maintain MHM, such as clean, private toilets, water and clean, reliable materials to absorb menses [7,20,21]. Lack of access to feminine hygiene products, female-friendly toilets, safe water and sanitation and soap as well as painkillers, can also act as barriers for females attending school while menstruating [13]. Where there are stigma and taboos associated with menstruation, concealment practices can also prevent effective MHM as girls internalise menstrual stigma [22–23].

The purpose of this quantitative, cross-sectional survey was to understand adolescents’ MHM practices, sources of information, attitudes towards menstruation, communication with parents and associated factors in urban and peri-urban areas in Lao PDR, a lower-middle-income country in South-East Asia. This contributes to the growing literature on MHM among adolescent in LMICs and more specifically within Lao PDR, which has a large population proportion of adolescents. According to the most recent census, adolescents (11–19 years) comprised 23% of the total population [24]. Little is known, however, about adolescent MHM. One study conducted in 2014 in rural areas of Savannakhet province in southern Lao PDR, which included females over 15 years of age, found many participants were unable to practice effective MHM due to the social determinants of health including lack of access to the toilets, water and sanitation and sanitary pads [25]

This paper contributes to the growing literature on MHM among adolescents in Lao PDR. Furthermore, young adolescent girls are typically under-prepared for menstruation at menarche [2], and most of the literature on adolescent MHM focuses on older adolescents in LMICs. In contrast to most studies, which focus on older adolescents and are school-based, meaning we know little about out-of-school adolescents, here we included in and out-of-school adolescents aged 11–19.

## Methods

This is a cross-sectional, descriptive study, conducted between November 2018 and May 2019. The sampling unit included secondary schools in Khammouane and Champasack provinces (in central and southern Lao PDR, respectively) and out-of-school adolescent girls in the same provinces. The higher secondary schools were all located in urban areas, while out-of-school participants were selected from villages in peri-urban areas. All participants were aged between 11 and 19 years and consented to participate.

### Sampling

In-school adolescents were selected from six secondary schools, (following permission from the school directors). The schools were all located in urban areas and selected using simple random sampling using a list of the 16 secondary schools in Khammouane and Champassak provinces. In each selected school, the list of students in each grade for the academic year 2018–2019 was prepared and simple-random sampling applied to identify potential participants.

For the out-of-school adolescents, a list of villages in each of the districts where the selected higher secondary schools were located was prepared. Based on the list of villages, five villages were selected using simple random sampling. In each of the selected villages, a list of adolescent girls was provided by the Village Head from which participants were selected using simple random sampling.

The sample size was determined using a formula for the estimation of single population proportion and based on the assumption of 95% CI, 5% margin of error, and estimating 50% of respondents had good MHM practices. To compensate for anticipated non-response, 13% of the determined sample was added to the calculated sample size with the final sample size calculated as 433. Of the total number of participants sampled, only 343 girls had reached menarche. Of these 343 participants, the number of in-school adolescent girls was 244 and out-of-school 99.

### Data collection

A predesigned and pretested questionnaire was developed based on literature to determine the level (good/poor) of MHM practices [e.g. 16,22]. The questionnaires were self-administered for in-school adolescents and administered in face-to-face interviews for out-of-school adolescents. The questionnaire consisted of two parts. One part collected information on the demographic characteristics of participants, including perceived socio-economic status and parent-adolescent discussion about menstruation while the other part related to menstrual hygiene practices. Questions included being absent from school/staying at home or abstaining from household work due to menstruation.

To assess MHM practices five binary yes/no questions were posed related to use of disposable pad/panty liner to manage menses, whether pads were changed as needed during bleeding, ability to wash or take a bath with soap as needed, whether participants were able to change pads in school whenever necessary and they were able to dispose of or dry the reusable pad in privacy. Each affirmative answer was coded as one and negative answers coded as zero, with the score for each question summed. Based on Bloom’s suggested cut-off point [26,27] a score of higher than 80% of correct answers was categorised as practicing ‘good’ MHM, whereas a score less than 80% was categorised as having ‘poor’ MHM practices.

### Data analysis

Data were entered on an ongoing basis into Epi data, so data quality could be checked throughout the data collection period. The statistical package STATA 15 was used to analyse the data. Adolescents were categorised into two age groups: 11–14 years and 15–19 years. Factors associated with MHM were examined using bivariate and multivariate analyses. Multivariate analysis was performed to identify factors associated with MHM. Only variables with p < 0.25 from the initial bivariate analyses were included in the model and steps were taken to control for confounding variables such as the socio-demographic characteristics of the participants. Backward stepwise elimination was applied to the multivariate analysis. Statistical significance was established at p < 0.05 and all tests were 2-tailed. Odds ratios and 95% confidence intervals were calculated.

### Ethical approval

The study received ethical approval from the Ethical Committee of the University of Health Sciences, Lao PDR No 061, dated 23/11/2018. Verbal consent was obtained with ethical approval before interviewing participants aged 18 years and over. For participants aged between 15 and 17 years, with ethical approval, the participant gave consent based on the assumption young people of this age group are competent (Gillick competency principle) and not wishing to insist on proxy consent when the young people were competent [28]. This is consistent with Lao family law which recognises young people aged 15–17 are able to provide informed consent [29]. Participants were, however, able to include another person, including a caregiver, in the consent process if they wished.

For all participants aged 11–14 years, assent was obtained from participants as well as from their guardians/parents. Anonymity, privacy and confidentiality were assured with the voluntary nature of participation and the right to withdraw at any time-stressed to participants.

## Results

### Socio-demographic characteristics of participants

A total of 343 adolescent girls from Khammouane and Champasack provinces who had reached menarche participated in the study, of which 99 were out-of-school (29.7%). The mean age of participants was 15.6 with a range of 11–19 years. The mean age of in-school adolescents was 15.4 years and 16.0 years for out-of-school adolescents. As seen in Table 1, there was a slightly higher number of younger aged participants (11–15 years) compared with than the older age (16–19 years) participants (51.9% and 48.1%, respectively). Almost all participants had some level of schooling with most of the in-school participants, in lower secondary school (43.7%). Most of the participants’ mothers and fathers were reported to have some level of education and were working at the time of the study. Based on participants’ subjective assessments, the majority placed their family in middle-level socio-economic status.

**Table 1.** Socio-demographic characteristic of 343 adolescent girls in Lao PDR.

	In-school		Out-of school		Total	
Variables	N = 244	%	N = 99	%	N = 343	%
Age Mean (SD) Range	15.4 (2.0) 12 to 19		16.0 (1.2) 11 to 19		15.6 (1.9) 11 to 19	
11–15 years	124	50.8	41	41.4	165	48.1
16–19 years	120	49.2	58	58.6	178	51.9
Total	244	100	99	100	343	100
**Education**						
Illiterate	0	0.0	3	3.0	3	0.9
Primary	0	0.0	17	17.2	17	5.0
Complete primary	0	0.0	30	30.3	30	8.7
Lower Secondary	111	45.5	39	39.4	150	43.7
Upper Secondary	130	53.3	9	9.1	139	40.5
College	1	0.4	0	0.0	1	0.3
No answer	2	0.8	1	1.0	3	0.9
Total	244	100	99	100	343	100
Which class/grade are you now at this school?						
Primary Grade 5	15	6.1	-	-	15	6.1
Lower Grade 1	25	10.2	-	-	25	10.2
Lower Grade 2	23	9.4	-	-	23	9.4
Lower Grade 3	45	18.4	-	-	45	18.4
Lower Grade 4	46	18.9	-	-	46	18.9
Lower Grade 5	35	14.3	-	-	35	14.3
Lower Grade 6	41	16.8	-	-	41	16.8
Lower Grade 7	2	0.8	-	-	2	0.8
Others	1	0.4	-	-	1	0.4
No answer	11	4.5	-	-	11	4.5
Total	244	100	-	-	244	100
What is your father’s highest level of school completed? (N = 317)						
Illiterate	1	0.4	2	2.2	3	0.9
Incomplete Primary	19	8.5	10	10.8	29	9.1
Complete primary	25	11.2	15	16.1	40	12.6
Lower Secondary	39	17.4	14	15.1	53	16.7
Upper Secondary	27	12.1	17	18.3	44	13.9
College	11	4.9	3	3.2	14	4.4
University	31	13.8	3	3.2	34	10.7
Unknown	65	29.0	27	29.0	92	29.0
No answer	6	2.7	2	2.2	8	2.5
Total	224	100	93	100	317	100
Does your father have a job? (N = 317)						
Yes	182	81.3	76	81.7	258	81.4
No	36	16.1	15	16.1	51	16.1
No answer	6	2.7	2	2.2	8	2.5
Total	224	100	93	100	317	100
If not, why does your father have not a job? (N = 51)						
He is sick or retired	4	11.1	0	0.0	4	7.8
He is looking for a job	5	13.9	0	0.0	5	9.8
He takes care of others or	17	47.2	6	40.0	23	45.1
Don’t know	9	25.0	6	40.0	15	29.4
Other	1	2.8	0	0.0	1	2.0
No answer	0	0.0	3	20.0	3	5.9
Total	36	100	15	100	51	100
What is the highest level of school of your mother has completed (N = 332)						
Illiterate	3	1.3	7	7.3	10	3.0
Primary	34	14.4	18	18.8	52	15.7
Complete primary	39	16.5	20	20.8	59	17.8
Lower Secondary	48	20.3	15	15.6	63	19.0
Upper Secondary	20	8.5	10	10.4	30	9.0
College	10	4.2	4	4.2	14	4.2
University	12	5.1	3	3.1	15	4.5
Unknown	66	28.0	19	19.8	85	25.6
No answer	4	1.7	0	0.0	4	1.2
Total	236	100	96	100	332	100
Does your mother have a job? (N = 332)						
Yes	158	66.9	75	78.1	233	70.2
No	73	30.9	20	20.8	93	28.0
No answer	5	2.1	1	1.0	6	1.8
Total	236	100	96	100	332	100
If not, why does your mother not have a job (N = 93)						
She is sick or retired	1	1.4	1	5.0	2	2.2
She is looking for a job	3	4.1	0	0.0	3	3.2
She takes care of others	50	68.5	9	45.0	59	63.4
Unknown	14	19.2	5	25.0	19	20.4
Other	3	4.1	3	15.0	6	6.5
No answer	2	2.7	2	10.0	4	4.3
Total	73	100	20	100	93	100
How do you rate the socio-economic status of your family*						
Rich	1	0.4	1	1.0	2	0.6
High Middle	4	1.6	1	1.0	5	1.5
Middle	227	93.0	87	87.9	314	91.5
Poor	8	3.3	10	10.1	18	5.2
Very poor	1	0.4	0	0.0	1	0.3
No answer	3	1.2	0	0.0	3	0.9
Total	244	100	99	100	343	100

*This is based on the perception of the girls of their family’s socio-economic status.

### Menstruation

Mean age at menarche was 12.7 years (SD 1.1) with the minimum age 10 years and the maximum 18 years. Around half of participants (48.1%) reported experiencing menarche by the age of 12 years while the remainder experienced menarche from 13 years onwards. There was no difference in age at menarche between in-school and out-of-school girls 12.5 (SD = 1.1) for in-school girls versus 13.0 (SD 1.3) for out-of-school girls. Of all participants, 84.3% reported menstruating every month while 15.2% reported menstruating occasionally., with 54.2% of girls saying their menstrual cycle was 14 days or less. Based on their subjective assessment, most participants felt their blood flow during menstruation was normal (78.7%) while 16.0% said they experienced a lot of bleeding.

Among the participants who reported experiencing symptoms before or after their period, the more commonly reported symptoms were irritability (31.0%), feeling heaviness of lower abdomen (23.7%), lethargy and tiredness (13.7%). Most reported abdominal pain during menstruation (89.3%) with 31.7% saying they took painkillers. Of the participants who experienced pain when menstruating, 25.7% said the pain interfered with their daily life. Table 2 shows participants’ reported experiences of menstruation.

**Table 2.** Experiences of menstruation among 343 adolescent girls in Lao PDR.

	In-school		Out-of school		Total	
Variables	N = 244	%	N = 99	%	N = 343	%
At what age did you have your first menstrual period? (n = 343)						
Mean (SD)	12.5 (1.1)		13.0 (1.3)		12.7 (1.1)	
Minimum, Maximum	10, 15		10, 18		10, 18	
10–12 years	129	52.9	36	36.4	165	48.1
13–18 years	115	47.1	63	63.6	178	51.9
Is your menstruation regular or coming every month?						
Yes	207	84.8	82	82.8	289	84.3
No	35	14.3	17	17.2	52	15.2
No answer	2	0.8	0	0.0	2	0.6
Total	244	100	99	100	343	100
What is your average menstrual cycle length?						
≤ 14	146	59.8	40	40.4	186	54.2
15–30 days	86	35.3	58	58.6	144	42.0
No answer	12	4.9	1	1.0	13	3.8
What is the amount of bleeding each time?						
Little	12	4.9	1	1.0	13	3.8
Normal	196	80.3	74	74.7	270	78.7
A Lot	31	12.7	24	24.2	55	16.0
No answer	5	2.0	0	0.0	5	1.5
Total	244	100	99	100	343	100
Do you have any symptoms before or after menstruation?						
Yes	195	79.9	76	76.8	271	79.0
No	44	18.0	23	23.2	67	19.5
No answer	5	2.0	0	0.0	5	1.5
Total	244	100	99	100	343	100
If you marked ‘Yes’, how often do you have the symptom?						
Every month	85	43.6	56	73.7	141	52.0
Most months	13	6.7	3	3.9	16	5.9
Occasionally	76	39.0	13	17.1	89	32.8
Rarely	19	9.7	3	3.9	22	8.1
No answer	2	1.0	1	1.3	3	1.1
Total	195	100	76	100	271	100
What kind of symptoms do you have? (multiple choices)						
Loss of/increased appetite	22	11.3	5	6.6	27	3.9
Depression	29	14.9	11	14.5	40	5.8
Irritability	152	77.9	63	82.9	215	31.0
Inability to concentrate on work	41	21.0	7	9.2	48	6.9
Lethargy and tiredness	72	36.9	23	30.3	95	13.7
Headache	43	22.1	10	13.2	53	7.6
Sleeplessness or increased sleep	28	14.4	10	13.2	38	5.5
Feeling heaviness of lower abdomen	117	60.0	47	61.8	164	23.7
Other	11	5.6	2	2.6	13	1.9
Total	515	264.1	178	234.2	693	100.0
Have you experienced menstrual pain?						
Yes	171	87.7	70	92.1	241	89.3
No	23	11.8	6	7.9	29	10.7
No answer	1	0.5	0	0.0	1	0.4
If you marked ‘Yes’, how was your menstrual pain in last 3 months?						
Little pain	121	70.8	58	82.9	179	74.3
Pain interfered with my daily life	41	24.0	10	14.3	51	21.2
Severe pain interfered with my daily life	9	5.2	2	2.8	11	4.5
Have you ever taken any painkiller?						
Yes	54	31.6	22	31.4	76	31.7
No	117	68.4	47	67.1	164	68.3
No answer	0	0.0	1	1.4	1	0.4
Were you familiar with menstruation before you got your first period?						
Yes	165	67.6	68	68.7	233	67.9
No	76	31.1	27	27.3	103	30.0
No answer	3	1.2	4	4.0	7	2.0

### Missing school/work or staying at home due to menstruation

Of the 343 participants, 26.5% said they had missed school or work because of their period, as shown in Table 3. The number of days participants reported staying at home during their last menstruation ranged from 1 to 23 days. The majority of the 91 girls who stayed at home (69.2%) reported taking 1 to 3 days off during their last menstruation. Of the possible health problems assessed in relation to missing school or work during their periods, the more common reasons for absence were cramps/headaches/pain (40.2%) and lack of spaces where they could rest in school (27.9%).

**Table 3.** Missing school or staying at home due to menstruation, among 343 adolescent girls in Lao PDR.

	In-school		Out-of school		Total		
Variables	N = 244	%	N = 99	%	N = 343	%	
Have you ever stayed home from school or work when you had your menstrual period?							
Yes	65	26.6	26	26.2	91	26.5	
No	174	71.3	71	71.8	245	71.5	
No answer	5	2.1	2	2.0	7	2.0	
During your last menstrual period, for how many days did you stay home from school/work?							
median, minimum, maximum	1, 1, 23		1, 1, 4		1, 1, 23		
1–3 days	45	69.2	18	69.2	63	69.2	
4–23 days	4	6.15	1	3.85	5	5.49	
No answer	16	24.6	7	26.9	23	25.3	
Why did you stay home during your last menstrual period?							
Cramps/Headache/Pain	113	46.3	25	25.2	138	40.2	
Diarrhoea	27	11.0	9	9.1	36	10.4	
Heavy Bleeding	4	1.6	1	1.0	5	1.4	
Lack of sanitary napkins or rags	5	2.0	0	0	5	1.4	
Lack of water/place to clean	3	1.2	14	14.1	17	4.9	
Lack of accommodation at school/work	61	25.0	35	35.3	96	27.9	
No answer	31	12.7	15	15.2	46	13.4	

### Parent-adolescent discussion about menstruation

Table 4 shows information related to parent-adolescent discussions about menstruation. Most girls (87.7%) had discussed menstruation with their mothers, mostly on more than one occasion. Few of the participants (8.4%), however, reported discussing menstruation with their fathers.

**Table 4.** Parent-adolescent discussion about menstruation among 343 adolescent girls in Lao PDR.

Variables	In-school		Out-of school		Total	
Fathers	N = 244	%	N = 99	%	N = 343	%
How often have you and your father talked about menstruation?						
Never	212	86.9	94	95.0	306	89.2
Once	9	3.7	1	1.0	10	2.9
A few times	17	7.0	2	2.0	19	5.5
Often	0	0.0	0	0.0	0	0.0
No answer	6	2.4	2	2.0	8	2.3
**Mothers**						
How often have you and your mother talked about menstruation?						
Never	21	8.6	17	17.2	38	11.1
Once	11	4.5	7	7.1	12	5.2
A few times	125	51.2	39	39.4	164	47.8
Often	83	34.0	36	36.3	119	34.7
No answer	4	1.7	0	0	4	1.2

### Menstrual hygiene management

Table 5 shows reported MHM practices during menstruation. Six in seven girls (85.4%) reported using disposable sanitary napkins, 84.3% said they were able to change their pad as needed and 90.1% said they were able to wash or take a bath with soap as needed. Only 63.0% said they were able to dispose of, or dry, reusable pads in privacy. The median monthly payment for sanitary products was 10,000 LAK with a minimum of 1,600 LAK and a maximum of 50,000 LAK (1$ = 8900 LAK), although there was one outlier at 80,000 LAK. Overall, 44.0% of participants indicated at least three correct answers on hygiene and sanitation practices, rated as good MHM.

**Table 5.** Menstrual Hygiene Management Practice among 343 adolescent girls in Lao PDR.

	In school		Out of school		Total	
	N = 244	%	N = 99	%	N = 343	%
What kind of absorbent pad or material do you use during menstruation?*						
Reusable cloth or towel	29	11.9	14	14.1	43	12.5
Disposable sanitary napkin	210	86.1	83	83.8	293	85.4
Traditional Lao skirt only	1	0.4	1	1.0	2	0.6
Other	1	0.4	1	1.0	2	0.6
No answer	3	1.2	0	0.0	3	0.9
Is the pad changed as needed? *						
Yes	208	85.2	81	81.8	289	84.3
No	20	8.2	15	15.2	35	10.2
No answer	16	6.6	3	3.0	19	5.5
Are you able to wash or take a bath with soap as needed? *						
Yes	214	87.7	95	96.0	309	90.1
Yes, but without soap	18	7.4	3	3.0	21	6.1
No	8	3.3	1	1.0	9	2.6
No answer	4	1.6	0	0.0	4	1.2
If no – what is the reason for not washing or taking a bath?						
Do not have enough water	1	12.5	0	0.0	1	11.1
Do not have a private bathroom	1	12.5	0	0.0	1	11.1
Do not feel the need to wash	2	25.0	0	0.0	2	22.2
Other	2	25.0	0	0.0	2	22.2
No answer	2	25.0	1	100.0	3	33.3
Are you able to dispose of a disposable napkin or dry a reusable pad in private?*						
Yes	141	57.8	75	75.8	216	63.0
No	96	39.3	24	24.2	120	35.0
No answer	7	2.9	0	0.0	7	2.0
If no, what was the reason?						
No facility to dispose privately	41	42.7	12	50.0	53	61.6
No private place to dry pad	7	7.3	1	4.2	8	9.3
Do not feel need for privacy	8	8.3	1	4.2	9	10.5
Other	14	14.6	2	8.3	16	18.6
No answer	26	27.1	8	33.3	34	39.5
How much do you spend on sanitary napkins per month? (1 $x00A0;= 8,900 LAK)						
Median	12,500		10,000		10,000	
Minimum, Maximum	2,000, 50,000		5,000, 40,000		2,000, 50,000	
Who pays for it?						
Myself	104	42.6	68	68.7	172	50.1
Mother	122	50.0	29	29.3	151	44.0
Father	2	0.8	1	1.0	3	0.9
Older Sister	3	1.2	1	1.0	4	1.2
Older Brother	0	0.0	0	0.0	0	0.0
Younger sibling	0	0.0	0	0.0	0	0.0
Other	2	0.8	0	0.0	2	0.6
No answer	11	4.5	0	0.0	11	3.2
Menstrual Hygiene Management Practice						
Good (score ≥80%)	103	42.2	48	48.5	151	44.0
Poor (score <80%)	141	57.8	51	51.5	192	56.0

### Factors affecting good practice of menstruation

Table 6 shows a multivariate logistic regression of MHM quality among participants with crude and adjusted odds ratios for good practice against a range of independent variables. Variables with a p-value <0.25 in the univariate analysis were entered into the multivariate model associated with the MHM practice were age group, education, father’s education and having mother-girl discussions about menstruation. Factors significantly associated with good MHM practice were older age 16–19 years (AOR 2.4; 95%CI 1.4 to 3.9); having primary education (AOR 0.4; 95%CI 0.2 to 0.9); father with high school education (AOR 2.1; 95%CI 1.0 to 4.1), and mother-girl discussion about menstruation (AOR 2.2; 95%CI 1.0 to 5.0).

**Table 6.** Multivariate logistic regression of menstrual hygiene management quality among 343 adolescent girls in Lao PDR, expressed as crude and adjusted odds ratios for good practice.

	N	%	COR	95%CI	P value	AOR	95%CI	P value
**Age(years)**								
11–15	55	33.3	ref			ref		
16–19	96	53.9	2.3	1.5 to 3.6	<0.001	2.4	1.4 to 3.9	0.001
**Age (SD) at menarche**	12.6 (1.181)		0.94	0.8 to 1.1	0.52			
**Ethnicity**								
Lao	145	43.5	ref					
Mone-Khmer	6	60.0	1.9	0.5 to 7.0	0.31			
**Education**								
Illiterate	27	54.0	ref			ref		
Primary	47	31.3	0.4	0.2 to 0.7	0.005	0.4	0.2 to 0.9	0.03
At least High School	77	53.9	1.0	0.5 to 1.9	0.99	0.9	0.5 to 1.9	0.90
**Currently studying**								
Yes (In school)	103	42.2	ref					
No (Out of school)	48	48.5	1.3	0.8 to 2.1	0.29			
**Father’s education**								
Primary or less	29	40.3	ref			ref		
High School	52	53.6	1.7	0.9 to 3.2	0.08	2.1	1.0 to 4.1	0.03
College or higher	61	41.2	1.0	0.6 to 1.8	0.89	1.4	0.7 to 2.6	0.29
**Father having a job**								
No	21	41.2	1					
Yes	117	45.4	1.2	0.6 to 2.2	0.58			
**Mother’s education**								
Primary or less	57	47.1	1					
High School	43	46.2	1.0	0.6 to 1.7	0.90			
College or higher	48	40.7	0.8	0.5 to 1.3	0.32			
**Mother having a job**								
No	41	44.1	1					
Yes	107	45.9	1.1	0.7 to 1.7	0.76			
**Father-girl discussion about menstruation**								
Never	139	44.3	1					
A few times	12	41.4	0.9	0.4 to 1.9	0.76			
**Mother-girl discussion about menstruation**								
Never	13	31.0	1			1		
A few times	138	45.9	1.9	0.9 to 3.8	0.072	2.2	1.0 to 5.0	0.049

## Discussion

To our knowledge, this is the first study in Lao PDR that specifically includes an examination of MHM practices in adolescents under 15 years of age. Overall, only 44% of girls scored 80% on good MHM practice. Other studies in LMICs have suggested generally inadequate MHM practices [13,15,26]. Nevertheless, based on a cumulative score for in-school and out-of-school girls many MHM practices were enacted correctly.

Slightly more out-of-school girls recorded good MHM scores compared with school-going girls, but this difference was not significant. The reason for the higher number of out-of-school girls being able to practice good MHM may be that compared to in-school adolescents, out-of-school girls are working or at home with better access to safe and private places to wash and change and dispose of sanitary pads. Being older (16–19 years) was associated with good MHM practice and is probably due to older girls having more experience and better opportunities to share information regarding menstrual hygiene compared with younger girls [23]. It may also be that the older girls had a greater degree of financial independence and were able to purchase hygiene supplies such as soap and sanitary pads themselves. The overall level of MHM practice, however, is of concern as suboptimal practice can contribute to unfavourable sexual and reproductive health outcomes as well as poorer academic performance and subsequent employment opportunities.

Providing adolescents with the information and tools they need for effective MHM practice before they reach menarche is critical for adolescents’ physical and mental health. Suboptimal MHM may relate to not having access to a safe and private place [1,13,16]. While knowledge is likely to be one reason for poor MHM practice, other reasons such as lack of access to the hardware of MHM including physical infrastructure, access to markets or cash, reduces adolescents’ capacity in upholding good hygienic practice, even for those who have the knowledge. Absences from school are likely to be due to discomfort or pain while menstruating but also due to poor facilities at school, such as safe and private latrines [7,25,30,32]. Over half of the included participants reported that they could not dispose of a used sanitary pad in private [7,19]. Embarrassment and social taboos may also help explain school absences and requires further qualitative research [32]. Education about MHM, ensuring access to accessible sanitary products, pain relief and adequate sanitary facilities could improve school attendance [19]. Further research is also needed on the link between menstruation and school attendance and performance [1].

In contrast with other studies in LMICs [8,25,33,34], the majority of adolescents reported using commercial sanitary pads and said they were able to change them and wash with soap as needed. The reason for good access to commercial sanitary pads may be in part because the adolescents in this study resided in urban and peri-urban areas with access to markets and, possibly, a cash income. Most other studies in LMICs on the other hand have been conducted in rural areas [33,34]. One study among urban adolescent girls in Udupi Taluk, India, however, also reported similar findings to the present study regarding the use of sanitary pads [35]. A better understanding of how adolescents pay for commercial sanitary pads is warranted. Research in western Kenya with adolescents has identified that some girls engage in transactional sex to obtain money to buy pads [10,36–37]. A cost-effective alternative to sanitary pads, due to their reusability, may be menstrual cups [39,40] These are found to be acceptable for school-going adolescents where there is adequate support and mentoring in their introduction to build girls’ confidence in their use [39].

We found that fathers’ level of education was associated with good practice. The reason for this is not clear, but it may be that fathers with higher education also had better incomes. Mother and daughter discussions about menstruation were also associated with good MHM practices. Mothers are generally the main source of information about menstruation, probably as they are often the closest to their daughters and have lived experiences of menarche and menstruation [7,10,33,41,42]. Regardless, some mothers may not be fully informed themselves of good MHM practices or feel uncomfortable discussing reproduction and menstruation [2,9,11].

The results of the study reveal a slightly earlier age at menarche in just over half of the study participants (10 to 12 years) than has been reported in other studies, although variations in age at menarche have been recorded within and between countries. The general trend, however, suggests age at menarche is declining in most countries [43]. In LMICs a downward trend has also been observed, with surveys showing girls in many countries are now starting their periods on average at 12.5 years or earlier [26,34,35]. These reductions may be attributed to improved nutrition as well as improved socio-economic status and better healthcare [18,43,44]. Some caution is needed in interpreting our result however as the exact age of onset of menarche could not be calculated due to lack of availability of accurate information or recall bias.

A limitation of this cross-sectional study is that it only provides a snapshot in time and does not provide information on cause and effect relationships between study variables. Furthermore, we used different data collection methods for the in-school and out-of-school adolescents (i.e. face-to-face administration for out-of-school adolescents due to lower literacy and self-administration for in-school adolescents). The use of these two different data capture methods means some caution is needed in interpreting differences between in-school and out-of-school girls. In addition, we relied on subjective assessments of family socio-economic positions rather a direct measurement.

Another limitation is we did not ask about participants’ knowledge of MHM practice and it is difficult therefore to know whether those who had less than optimal practice were unaware of how to maintain good menstrual hygiene, or simply did not have access to the resources (such as sanitary pads or a toilet with privacy and adequate access to water and cleaning materials) needed. Menstrual cups are now being used in many countries and in several studies but were not included in our questionnaire and further research could also look at the availability and acceptability of menstrual cups in Lao PDR [39]. These results however open possibilities for further research to understand the knowledge gaps and freedoms needed to maintain a good level of hygiene and health, enabling more tailored interventions. Specifically, we suggest studies into barriers to good menstruation practices, including qualitative studies into taboos around menstruation, that consider ethnic and geographical diversity, and studies into knowledge of good menstruation practices.

## Conclusion

Inadequate MHM affects adolescent girls’ dignity, health and well-being. In this study, not all participants had the freedom to perform optimal menstrual hygiene. While sanitary pads seem reasonably accessible in helping adolescent girls manage their periods, improved safe, private, clean, and easily accessible water, sanitation, and disposal facilities are needed for women and girls to protect their menstrual health. Being able to practice good MHM is also important in being able to participate fully in education and work, although the direct economic and educational impacts related to good MHM practices require further research. There is also a need to design acceptable awareness creation and advocacy programs for adolescent schoolgirls and broader society to enable adolescents to uphold good hygienic practice.

## Paper context

Menarche symbols the beginning of menstrual cycling and the reproductive phase of a woman’s life. Inadequate menstrual hygiene, however, can have negative impacts on girl’s reproductive health and socio-economic outcomes. This study in Lao PDR, found not everyone can practice good menstrual hygiene due to inadequate access to access the hardware (e.g. toilets, water) needed for good practice. Increased access to safe water and sanitation and knowledge is needed to enable effective menstrual hygiene management.
